# 4-Bromo-2-{(*E*)-[(3,4-dimethyl­phen­yl)imino]­meth­yl}phenol

**DOI:** 10.1107/S1600536812035635

**Published:** 2012-08-23

**Authors:** M. Nawaz Tahir, Abdul Haleem Khan, Muhammad Ilyas Tariq, Ishtiaq Hussain, Muhammad Shafiq

**Affiliations:** a Department of Physics, University of Sargodha, Sargodha, Pakistan; b Department of Pharmacy Services, Jinnah Hospital, Lahore, Pakistan; c Department of Chemistry, University of Sargodha, Sargodha, Pakistan; d Department of Chemistry, Government College University, Faisalabad 38000, Pakistan

## Abstract

In the title compound, C_15_H_14_BrNO, the dihedral angle between the aromatic rings is 4.10 (11)° and the mol­ecule is close to planar (r.m.s. deviation for the non-H atoms = 0.053 Å). An intra­molecular O—H⋯N hydrogen bond closes an *S*(6) ring. In the crystal, very weak C—H⋯π inter­actions are observed.

## Related literature

For related structures, see: Unver *et al.* (2010 ▶). For graph-set notation, see: Bernstein *et al.* (1995 ▶).
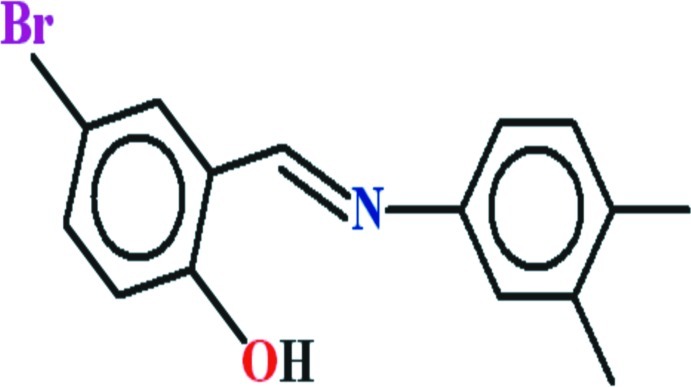


## Experimental

### 

#### Crystal data

C_15_H_14_BrNO
*M* *_r_* = 304.18
Monoclinic, 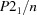

*a* = 12.2633 (10) Å
*b* = 7.4805 (6) Å
*c* = 14.5767 (11) Å
β = 101.576 (4)°
*V* = 1310.00 (18) Å^3^
*Z* = 4
Mo *K*α radiation
μ = 3.13 mm^−1^
*T* = 296 K
0.30 × 0.25 × 0.22 mm

#### Data collection

Bruker Kappa APEXII CCD diffractometer
Absorption correction: multi-scan (*SADABS*; Bruker, 2005 ▶) *T*_min_ = 0.454, *T*_max_ = 0.546
9488 measured reflections
2545 independent reflections
1931 reflections with *I* > 2σ(*I*)
*R*_int_ = 0.026

#### Refinement

*R*[*F* ^2^ > 2σ(*F* ^2^)] = 0.028
*wR*(*F* ^2^) = 0.073
*S* = 1.04
2545 reflections
166 parameters
H-atom parameters constrained
Δρ_max_ = 0.30 e Å^−3^
Δρ_min_ = −0.34 e Å^−3^

### 

Data collection: *APEX2* (Bruker, 2007 ▶); cell refinement: *SAINT* (Bruker, 2007 ▶); data reduction: *SAINT*; program(s) used to solve structure: *SHELXS97* (Sheldrick, 2008 ▶); program(s) used to refine structure: *SHELXL97* (Sheldrick, 2008 ▶); molecular graphics: *ORTEP-3 for Windows* (Farrugia, 1997 ▶) and *PLATON* (Spek, 2009 ▶); software used to prepare material for publication: *WinGX* (Farrugia, 1999 ▶) and *PLATON*.

## Supplementary Material

Crystal structure: contains datablock(s) global, I. DOI: 10.1107/S1600536812035635/hb6936sup1.cif

Structure factors: contains datablock(s) I. DOI: 10.1107/S1600536812035635/hb6936Isup2.hkl

Supplementary material file. DOI: 10.1107/S1600536812035635/hb6936Isup3.cml

Additional supplementary materials:  crystallographic information; 3D view; checkCIF report

## Figures and Tables

**Table 1:** Hydrogen-bond geometry (Å, °) *Cg*1 is the centroid of the C1–C6 benzene ring.

*D*—H⋯*A*	*D*—H	H⋯*A*	*D*⋯*A*	*D*—H⋯*A*
O1—H1⋯N1	0.82	1.87	2.601 (2)	147
C7—H7*B*⋯*Cg*1^i^	0.96	2.95	3.668 (3)	133
C12—H12⋯*Cg*1^ii^	0.93	2.96	3.612 (2)	128

Symmetry codes: (i) 
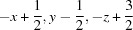
; (ii) 
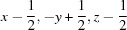
.

**Fig. 1.**
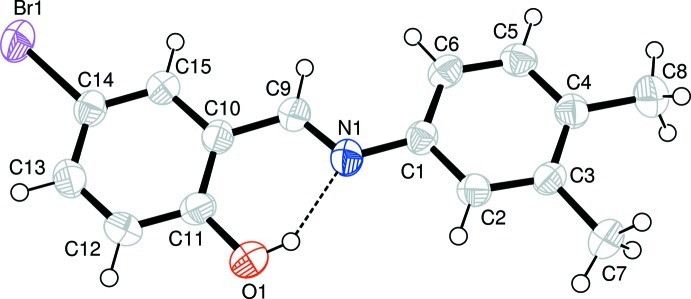
View of the title compound with displacement ellipsoids drawn at the 50% probability level. The dotted line represents the intramolecular hydrogen bond.

## supplementary crystallographic information

### Comment

The title compound, (Fig. 1) has been synthesized as a possible ligand for forming different metal complexes. its sturcture is now described. The crystal structures of 3,4-dimethyl-*N*-(3-nitrobenzylidene)aniline and 3,4-dimethyl-*N*-(4-nitrobenzylidene) aniline (Unver *et al.*, 2010) have been published which are related to the title compound.

The title compound is almost planar with r.m.s. deviation of 0.0487 Å, with maximum deviation of 0.1043 (11) Å for Br atom from the mean square plane. There exist intramolecular H-bonding of O—H···N type with *S*(6) ring motif (Bernstein *et al.*, 1995). There exist weak C—H···π interactions (Table 1) in the crystal.

### Experimental

Equimolar quantities of 3,4-dimethylaniline and 5-bromosalicylaldehyde were refluxed in methanol along with few drops of acetic acid as catalyst for 1 h. The solution was kept at room temperature which affoarded yellow prisms of the title compound after two days.

### Refinement

The H-atoms were positioned geometrically (C–H = 0.93–0.96 Å, O—H = 0.82 Å) and refined as riding with *U*_iso_(H) = *xU*_eq_(C, O) where *x* = 1.5 for hydroxy & methyl and *x* = 1.2 for other H-atoms.

### Crystal data

**Table:**

C_15_H_14_BrNO	*F*(000) = 616
*M**_r_* = 304.18	*D*_x_ = 1.542 Mg m^−^^3^
Monoclinic, *P*2_1_/*n*	Mo *K*α radiation, λ = 0.71073 Å
Hall symbol: -P 2yn	Cell parameters from 1931 reflections
*a* = 12.2633 (10) Å	θ = 2.0–26.0°
*b* = 7.4805 (6) Å	µ = 3.13 mm^−^^1^
*c* = 14.5767 (11) Å	*T* = 296 K
β = 101.576 (4)°	Prism, yellow
*V* = 1310.00 (18) Å^3^	0.30 × 0.25 × 0.22 mm
*Z* = 4	

### Data collection

**Table:**

Bruker Kappa APEXII CCD diffractometer	2545 independent reflections
Radiation source: fine-focus sealed tube	1931 reflections with *I* > 2σ(*I*)
Graphite monochromator	*R*_int_ = 0.026
Detector resolution: 8.00 pixels mm^-1^	θ_max_ = 26.0°, θ_min_ = 2.0°
ω scans	*h* = −10→15
Absorption correction: multi-scan (*SADABS*; Bruker, 2005)	*k* = −9→8
*T*_min_ = 0.454, *T*_max_ = 0.546	*l* = −17→17
9488 measured reflections	

### Refinement

**Table:**

Refinement on *F*^2^	Primary atom site location: structure-invariant direct methods
Least-squares matrix: full	Secondary atom site location: difference Fourier map
*R*[*F*^2^ > 2σ(*F*^2^)] = 0.028	Hydrogen site location: inferred from neighbouring sites
*wR*(*F*^2^) = 0.073	H-atom parameters constrained
*S* = 1.04	*w* = 1/[σ^2^(*F*_o_^2^) + (0.0371*P*)^2^ + 0.1325*P*] where *P* = (*F*_o_^2^ + 2*F*_c_^2^)/3
2545 reflections	(Δ/σ)_max_ = 0.001
166 parameters	Δρ_max_ = 0.30 e Å^−^^3^
0 restraints	Δρ_min_ = −0.34 e Å^−^^3^

### Special details

**Table:**

Geometry. Bond distances, angles *etc*. have been calculated using the rounded fractional coordinates. All su's are estimated from the variances of the (full) variance-covariance matrix. The cell e.s.d.'s are taken into account in the estimation of distances, angles and torsion angles
Refinement. Refinement of *F*^2^ against ALL reflections. The weighted *R*-factor *wR* and goodness of fit *S* are based on *F*^2^, conventional *R*-factors *R* are based on *F*, with *F* set to zero for negative *F*^2^. The threshold expression of *F*^2^ > σ(*F*^2^) is used only for calculating *R*-factors(gt) *etc*. and is not relevant to the choice of reflections for refinement. *R*-factors based on *F*^2^ are statistically about twice as large as those based on *F*, and *R*- factors based on ALL data will be even larger.

### Fractional atomic coordinates and isotropic or equivalent isotropic displacement parameters (Å^2^)

**Table:**

	*x*	*y*	*z*	*U*_iso_*/*U*_eq_	
Br1	0.18394 (2)	0.52508 (4)	0.05809 (2)	0.0611 (1)	
O1	0.04873 (13)	0.3558 (3)	0.42252 (10)	0.0649 (7)	
N1	0.23707 (15)	0.4902 (2)	0.50899 (13)	0.0433 (6)	
C1	0.30890 (17)	0.5327 (3)	0.59525 (15)	0.0377 (7)	
C2	0.26822 (18)	0.4991 (3)	0.67585 (15)	0.0402 (7)	
C3	0.32937 (18)	0.5327 (3)	0.76461 (15)	0.0386 (7)	
C4	0.43689 (17)	0.6028 (3)	0.77377 (14)	0.0413 (7)	
C5	0.47658 (17)	0.6376 (3)	0.69271 (14)	0.0445 (8)	
C6	0.41546 (16)	0.6041 (3)	0.60481 (15)	0.0437 (7)	
C7	0.2812 (2)	0.4915 (3)	0.85007 (17)	0.0559 (9)	
C8	0.50899 (19)	0.6368 (3)	0.86868 (15)	0.0595 (9)	
C9	0.26191 (19)	0.5199 (3)	0.42938 (15)	0.0411 (7)	
C10	0.18689 (18)	0.4725 (3)	0.34246 (15)	0.0384 (7)	
C11	0.08370 (17)	0.3910 (3)	0.34249 (15)	0.0442 (7)	
C12	0.01435 (18)	0.3464 (3)	0.25816 (16)	0.0495 (8)	
C13	0.04434 (18)	0.3849 (3)	0.17425 (15)	0.0462 (7)	
C14	0.14496 (18)	0.4672 (3)	0.17412 (15)	0.0407 (7)	
C15	0.21649 (18)	0.5098 (3)	0.25677 (15)	0.0406 (7)	
H1	0.09704	0.38542	0.46739	0.0974*	
H2	0.19695	0.45203	0.66987	0.0483*	
H5	0.54756	0.68561	0.69831	0.0534*	
H6	0.44489	0.62870	0.55214	0.0524*	
H7A	0.32881	0.40851	0.88946	0.0838*	
H7B	0.20851	0.44010	0.83078	0.0838*	
H7C	0.27580	0.59987	0.88418	0.0838*	
H8A	0.47412	0.72447	0.90136	0.0893*	
H8B	0.58039	0.68003	0.86120	0.0893*	
H8C	0.51838	0.52756	0.90395	0.0893*	
H9	0.32978	0.57328	0.42700	0.0493*	
H12	−0.05316	0.28997	0.25835	0.0594*	
H13	−0.00291	0.35554	0.11803	0.0554*	
H15	0.28458	0.56350	0.25552	0.0487*	

### Atomic displacement parameters (Å^2^)

**Table:**

	*U*^11^	*U*^22^	*U*^33^	*U*^12^	*U*^13^	*U*^23^
Br1	0.0679 (2)	0.0809 (2)	0.0349 (2)	−0.0060 (1)	0.0114 (1)	0.0038 (1)
O1	0.0642 (11)	0.0893 (14)	0.0442 (9)	−0.0253 (10)	0.0179 (8)	0.0017 (9)
N1	0.0464 (10)	0.0475 (11)	0.0355 (10)	−0.0011 (8)	0.0073 (8)	−0.0028 (8)
C1	0.0431 (12)	0.0358 (11)	0.0349 (11)	0.0024 (9)	0.0094 (9)	−0.0005 (9)
C2	0.0409 (12)	0.0401 (13)	0.0407 (12)	−0.0035 (9)	0.0105 (10)	−0.0015 (9)
C3	0.0461 (12)	0.0359 (12)	0.0359 (11)	0.0004 (10)	0.0135 (9)	0.0012 (9)
C4	0.0468 (12)	0.0348 (12)	0.0411 (12)	0.0005 (10)	0.0059 (10)	0.0003 (10)
C5	0.0379 (12)	0.0455 (14)	0.0498 (14)	−0.0034 (10)	0.0080 (10)	0.0022 (10)
C6	0.0436 (13)	0.0494 (13)	0.0413 (12)	−0.0001 (10)	0.0160 (10)	0.0036 (10)
C7	0.0664 (16)	0.0653 (17)	0.0388 (13)	−0.0078 (12)	0.0173 (12)	0.0006 (10)
C8	0.0621 (15)	0.0662 (18)	0.0460 (14)	−0.0063 (13)	0.0005 (12)	−0.0015 (12)
C9	0.0439 (12)	0.0426 (13)	0.0371 (12)	−0.0022 (10)	0.0091 (9)	−0.0008 (9)
C10	0.0435 (12)	0.0362 (12)	0.0352 (12)	0.0007 (9)	0.0074 (9)	0.0001 (9)
C11	0.0487 (13)	0.0442 (13)	0.0420 (12)	−0.0054 (11)	0.0146 (10)	0.0032 (10)
C12	0.0444 (13)	0.0498 (15)	0.0529 (14)	−0.0121 (10)	0.0063 (11)	−0.0003 (11)
C13	0.0487 (13)	0.0445 (13)	0.0414 (12)	−0.0010 (11)	−0.0006 (10)	−0.0014 (10)
C14	0.0460 (13)	0.0399 (12)	0.0365 (12)	0.0045 (10)	0.0089 (10)	−0.0001 (9)
C15	0.0414 (12)	0.0416 (13)	0.0391 (12)	−0.0006 (9)	0.0091 (10)	0.0009 (9)

### Geometric parameters (Å, º)

**Table:**

Br1—C14	1.898 (2)	C12—C13	1.377 (3)
O1—C11	1.347 (3)	C13—C14	1.379 (3)
O1—H1	0.8200	C14—C15	1.378 (3)
N1—C1	1.419 (3)	C2—H2	0.9300
N1—C9	1.277 (3)	C5—H5	0.9300
C1—C6	1.393 (3)	C6—H6	0.9300
C1—C2	1.388 (3)	C7—H7A	0.9600
C2—C3	1.382 (3)	C7—H7B	0.9600
C3—C4	1.400 (3)	C7—H7C	0.9600
C3—C7	1.514 (3)	C8—H8A	0.9600
C4—C8	1.507 (3)	C8—H8B	0.9600
C4—C5	1.390 (3)	C8—H8C	0.9600
C5—C6	1.372 (3)	C9—H9	0.9300
C9—C10	1.452 (3)	C12—H12	0.9300
C10—C15	1.397 (3)	C13—H13	0.9300
C10—C11	1.405 (3)	C15—H15	0.9300
C11—C12	1.388 (3)		
			
C11—O1—H1	109.00	C1—C2—H2	119.00
C1—N1—C9	123.19 (19)	C3—C2—H2	119.00
N1—C1—C6	125.30 (19)	C4—C5—H5	119.00
C2—C1—C6	118.3 (2)	C6—C5—H5	119.00
N1—C1—C2	116.41 (19)	C1—C6—H6	120.00
C1—C2—C3	122.7 (2)	C5—C6—H6	120.00
C2—C3—C7	120.4 (2)	C3—C7—H7A	109.00
C4—C3—C7	120.89 (19)	C3—C7—H7B	109.00
C2—C3—C4	118.8 (2)	C3—C7—H7C	109.00
C3—C4—C8	121.25 (19)	H7A—C7—H7B	110.00
C5—C4—C8	120.53 (19)	H7A—C7—H7C	109.00
C3—C4—C5	118.22 (19)	H7B—C7—H7C	109.00
C4—C5—C6	122.8 (2)	C4—C8—H8A	109.00
C1—C6—C5	119.3 (2)	C4—C8—H8B	109.00
N1—C9—C10	121.7 (2)	C4—C8—H8C	109.00
C9—C10—C11	121.2 (2)	H8A—C8—H8B	109.00
C9—C10—C15	119.9 (2)	H8A—C8—H8C	109.00
C11—C10—C15	118.9 (2)	H8B—C8—H8C	109.00
O1—C11—C10	121.90 (19)	N1—C9—H9	119.00
O1—C11—C12	118.36 (19)	C10—C9—H9	119.00
C10—C11—C12	119.7 (2)	C11—C12—H12	120.00
C11—C12—C13	120.8 (2)	C13—C12—H12	120.00
C12—C13—C14	119.6 (2)	C12—C13—H13	120.00
Br1—C14—C13	119.24 (16)	C14—C13—H13	120.00
C13—C14—C15	121.0 (2)	C10—C15—H15	120.00
Br1—C14—C15	119.77 (17)	C14—C15—H15	120.00
C10—C15—C14	120.1 (2)		
			
C9—N1—C1—C2	177.4 (2)	N1—C9—C10—C11	−0.3 (3)
C9—N1—C1—C6	−2.7 (3)	N1—C9—C10—C15	179.0 (2)
C1—N1—C9—C10	179.2 (2)	C9—C10—C11—O1	1.1 (3)
N1—C1—C2—C3	179.5 (2)	C9—C10—C11—C12	−179.6 (2)
C6—C1—C2—C3	−0.4 (3)	C15—C10—C11—O1	−178.2 (2)
N1—C1—C6—C5	−179.5 (2)	C15—C10—C11—C12	1.1 (3)
C2—C1—C6—C5	0.4 (3)	C9—C10—C15—C14	−179.2 (2)
C1—C2—C3—C4	−0.3 (3)	C11—C10—C15—C14	0.2 (3)
C1—C2—C3—C7	−179.3 (2)	O1—C11—C12—C13	177.8 (2)
C2—C3—C4—C5	0.8 (3)	C10—C11—C12—C13	−1.5 (3)
C2—C3—C4—C8	−178.0 (2)	C11—C12—C13—C14	0.6 (3)
C7—C3—C4—C5	179.9 (2)	C12—C13—C14—Br1	−178.45 (17)
C7—C3—C4—C8	1.0 (3)	C12—C13—C14—C15	0.7 (3)
C3—C4—C5—C6	−0.8 (3)	Br1—C14—C15—C10	178.07 (17)
C8—C4—C5—C6	178.1 (2)	C13—C14—C15—C10	−1.1 (3)
C4—C5—C6—C1	0.2 (4)		

### Hydrogen-bond geometry (Å, º)

Cg1 is the centroid of the C1–C6 benzene ring.

**Table:**

*D*—H···*A*	*D*—H	H···*A*	*D*···*A*	*D*—H···*A*
O1—H1···N1	0.82	1.87	2.601 (2)	147
C7—H7*B*···*Cg*1^i^	0.96	2.95	3.668 (3)	133
C12—H12···*Cg*1^ii^	0.93	2.96	3.612 (2)	128

Symmetry codes: (i) −*x*+1/2, *y*−1/2, −*z*+3/2; (ii) *x*−1/2, −*y*+1/2, *z*−1/2.
